# The effectiveness of emergency nurse practitioner service in the management of patients presenting to rural hospitals with chest pain: a multisite prospective longitudinal nested cohort study

**DOI:** 10.1186/s12913-017-2395-9

**Published:** 2017-06-27

**Authors:** Tina E. Roche, Glenn Gardner, Leanne Jack

**Affiliations:** 1 School of Nursing, Queensland University of Technology, Victoria Park Road, Kelvin Grove, QLD 4059 Australia; 2 Queensland University of Technology, Institute of Health and Biomedical Innovation Victoria Park Road, Victoria Park Road, Kelvin Grove, QLD 4059 Australia; 3 Queensland University of Technology, School of Nursing Victoria Park Road, Kelvin Grove, QLD 4059 Australia; 4 Emergency Department, Stanthorpe Health Services, PO Box 273, Stanthorpe, QLD 4380 Australia

**Keywords:** Rural health services, Chest pain, Emergency treatment, Patient satisfaction, Quality of care, Quality of life, Nested cohort, Cohort, Nurse practitioner, Adherence to guidelines

## Abstract

**Background:**

Health reforms in service improvement have included the use of nurse practitioners. In rural emergency departments, nurse practitioners work to the full scope of their expanded role across all patient acuities including those presenting with undifferentiated chest pain. Currently, there is a paucity of evidence regarding the effectiveness of emergency nurse practitioner service in rural emergency departments. Inquiry into the safety and quality of the service, particularly regarding the management of complex conditions is a priority to ensure that this service improvement model meets health care needs of rural communities.

**Methods:**

This study used a prospective, longitudinal nested cohort study of rural emergency departments in Queensland, Australia. Sixty-one consecutive adult patients with chest pain who presented between November 2014 and February 2016 were recruited into the study cohort. A nested cohort of 41 participants with suspected or confirmed acute coronary syndrome were identified. The primary outcome was adherence to guidelines and diagnostic accuracy of electrocardiograph interpretation for the nested cohort. Secondary outcomes included service indicators of waiting times, diagnostic accuracy as measured by unplanned representation rates, satisfaction with care, quality-of-life, and functional status. Data were examined and compared for differences for participants managed by emergency nurse practitioners and those managed in the standard model of care.

**Results:**

The median waiting time was 8.0 min (IQR 20) and length-of-stay was 100.0 min (IQR 64). Participants were 2.4 times more likely to have an unplanned representation if managed by the standard service model. The majority of participants (91.5%) were highly satisfied with the care that they received, which was maintained at 30-day follow-up measurement. In the evaluation of quality of life and functional status, summary scores for the SF-12 were comparable with previous studies. No differences were demonstrated between service models.

**Conclusions:**

There was a high level of adherence to clinical guidelines for the emergency nurse practitioner service model and a concomitant high level of diagnostic accuracy. Nurse practitioner service demonstrated comparable effectiveness to that of the standard care model in the evaluation of the service indicators and patient reported outcomes. These findings provide a foundation for the beginning evaluation of rural emergency nurse practitioner service in the delivery of safe and effective beyond the setting of minor injury and illness presentations.

**Trial registration:**

Australian New Zealand Clinical Trials Registry, ACTRN12616000823471 (Retrospectively registered).

**Electronic supplementary material:**

The online version of this article (doi:10.1186/s12913-017-2395-9) contains supplementary material, which is available to authorized users.

## Background

Health inequalities for people living in rural communities are well reported [1]. They are more likely to have shorter lives, increased risk factors and higher rates of chronic disease [2] which, when combined with lower access to health care, is likely to contribute to poorer health outcomes [1]. The rural context impacts on the capacity of health services to provide care.

In Australia there are twice as many rural hospital-based emergency facilities as there are metropolitan emergency departments [3], but there are lower numbers of health professionals and most health services do not employ dedicated emergency department staff. Additionally, health service usage differs between rural and metropolitan locations due in part to limited access to general practitioner consultations and higher rates of admission to hospital [4].

In response to the unique challenges for rural health services there has been a call for health reforms in service improvement by using new care models for the delivery of effective, appropriate and sustainable clinical care [5]. A range of service and workforce models have been implemented, including the use of nurse practitioners, as a strategy to improve access, efficiency and quality of care for patients [6].

The nurse practitioner service model is one of the most important developments in nursing in recent times, providing opportunity for significant reform in healthcare [7]. The International Council of Nurses define nurse practitioners as registered nurses who possess expert knowledge, complex decision-making skills and clinical competence [8] with legislated extensions for expanded practice including diagnosis, prescribing and referral. There is an abundance of literature that evidences the success of emergency nurse practitioner service both in Australia and internationally. Three systematic reviews have synthesised the evidence regarding the effectiveness of the emergency nurse practitioner service [9–11] finding a positive impact on patient satisfaction, waiting times and quality of care. However, the validity of these findings is open to a degree of criticism. The comparator against which the service was evaluated was almost invariably a medical practitioner, with a reliance on comparisons with junior doctors who do not share the advanced skills or practice privileges afforded to emergency nurse practitioners. Another limitation of these studies was a lack of consistency in the clinical skills of the services studied, because of considerable variance in the definitions and scope of practice internationally. Much of the research has used nurses who were not practicing to the full scope of the role, for example nurse practitioners in training.

Currently, there are around 1300 endorsed nurse practitioners in Australia working across a variety of specialty areas [12], with emergency being the single largest specialty group [13]. The initial impetus for this service model was to improve access and equity of care for emergency department patients who were experiencing long waiting times, with excessive times for patient management, diagnosis and discharge [14]. The most common emergency department presentations affected by these service issues were those with minor illness and injury. Accordingly, these presentations were the early focus of emergency nurse practitioner service, especially in metropolitan settings.

Not-with-standing the challenges, the rural environment presents many opportunities for innovation. Emergency nurse practitioners are now utilised as a service model in 38% of rural emergency departments [15] and nurse practitioners in these settings work to the full scope of their expanded role across all patient acuities including those presenting with undifferentiated chest pain.

Chest pain is a presentation of significance to emergency departments, representing 5–10% of all Australian annual patient presentations [16, 17]. Chest pain is symptomatic of many aetiologies, one of which is acute coronary syndrome. This encompasses a broad spectrum of clinical presentations that includes acute myocardial infarction which is the leading cause of sudden death in the Australian population [18].

Whilst chest pain is a characteristic of acute coronary syndrome, the majority of patients with chest pain are ultimately found to have non-cardiac diagnoses [16, 19–21]. Not-with-standing the diagnostic outcome, there are considerable costs to health services in evaluating patients who are experiencing chest pain. In the context of increasing health service demand, the challenge for clinicians, including nurse practitioners, in caring for this patient cohort is balancing risk and resources to determine an appropriate pathway of care and emergency department disposition [22].

Despite increasing use of the nurse practitioner service model, there is a paucity of evidence that is reported in the national and international literature regarding the safety and quality of the service. Robust review of current literature reveals that no experimental or observational studies have been published that specifically focus on evaluation of the safety and effectiveness of the nurse practitioner service model in the rural context. Clearly then, in acknowledging the paucity of evidence, there is a requirement to evaluate the quality of health care for those patients presenting to rural emergency departments with a complex and significant health care complaint.

## Methods

The aims of this study were to:

1. Examine the safety and quality of emergency nurse practitioner service in the provision of care in the rural emergency environment; and,
2. Evaluate the effectiveness of the emergency model in the management of patients presenting to emergency with undifferentiated chest pain.

### Study design and setting

The **Ma**naging chest **P**ain in **R**ural **E**mergency **D**epartments (MaP-RED) study was a prospective multicentre longitudinal nested cohort design. The study population was recruited from three rural emergency departments in Queensland, Australia. The study sites had similar service capabilities, no specialist cardiac services and were all located more than 150 km from the closest cardiac interventional hospital. All sites had both emergency nurse practitioners and medical officers providing management of patients presenting with undifferentiated chest pain.

### Participants and recruitment

Participants were enrolled consecutively between November 2014 and February 2016, at three rural emergency departments. Due to logistical factors, enrolment did not start and finish at the same time at each site. Whilst multisite human research ethics committee approval was granted, individual site specific approvals were protracted leading to delays in commencement at individual sites. Criteria for inclusion included patients with atraumatic chest pain who were at least 18 years old and able to provide informed consent (or have a legally acceptable representative). Patients who met inclusion criteria were identified by the triage nurse or treating clinician and invited to participate in the study. Participants were recruited to the study at the occasion-of-service in the emergency department. All participants provided written informed consent. Using the discharge diagnosis completed by the treating clinician in the Emergency Department Information System (EDIS™), a computerised management program used at each research site, a nested cohort was identified that consisted of patients with suspected or confirmed acute coronary syndrome. Specific EDIS™ discharge diagnoses included, but were not limited to, possible cardiac chest pain, angina pectoris, acute coronary syndrome and myocardial infarction.

Additionally, each nurse practitioner from the three study sites were invited to participate in the study. At the commencement of research for each site, informed consent was obtained from nurse practitioner participants for inclusion in the study.

### Data collection

Data were collected from patient participants at baseline and included demographic and clinical data. A self-administered patient questionnaire that measured patient-reported outcomes including satisfaction, quality-of-life and functional status was completed at baseline. Follow-up measurement occurred 30-days after the index presentation. Patient participant questionnaire developed for use in this study are available for review as Additional files 1, 2 and 3. Data were collected to examine for unplanned representation within seven-days of the occasion-of-service. This study was subsequently retrospectively registered with the Australian New Zealand Clinical Trials Registry, ACTRN12616000823471.

### Variables

The independent variable was the clinician service model involved in management of the patient. For study purposes, the models were operationally defined as:

- The emergency nurse practitioner service model that included the delivery and coordination of care in the diagnosis, investigation, therapeutic treatment (including prescribing of medications and technical interventions) and referral for patients with undifferentiated chest pain, or
- The standard care model was similar but delivered and coordinated by a medical officer.

In both models, all clinicians worked collaboratively and within their scope of practice. Emergency department nursing staff assisted the clinicians in providing care to the patient. There was no allocation of intervention; rather the care delivery model followed the standard method of patient allocation. Patients were treated in order of clinical urgency, the next available clinician (emergency nurse practitioner or medical officer) provided care as per the Australasian Triage Scale (ATS) allocation. The ATS is an indicator of clinical urgency where a number corresponds to the recommended timeframe in which a patient should receive treatment; a score of “1” indicates those patients with the most urgent needs through to a score of “5” representing patients with stable, minor symptoms or concerns [23].

Dependent variables were:

(i) Adherence to evidence-based guidelines for the management of suspected or confirmed acute coronary syndrome,
(ii) Diagnostic accuracy of electrocardiograph interpretation,
(iii) Service indicators of waiting times, length-of-stay and did-not-wait rates,
(iv) Diagnostic accuracy as measured by unplanned representation rates,
(v) Satisfaction with care; and,
(vi) Quality-of-life and functional status.

These variables were studied and compared across the two clinician service model groups.

### Data sources/measurement

After informed consent was obtained, baseline data relating to demographic and clinical indicators were collected prospectively by research assistants using a specifically designed data abstraction tool. At the completion of the occasion-of-service, all study participants completed a self-administered questionnaire that measured patient-reported outcomes including satisfaction, quality-of-life and functional status. Data for unplanned representations to the emergency department were collected seven-days after the index presentation. Follow-up questionnaires were posted to all study participants at 30-days after the initial emergency department presentation.

Two tested and validated tools were used to develop these participant questionnaires. The first tool used was a modified AUSPRAC patient outcomes scale [24] that incorporated multiple validated and tested tools. We used the components of satisfaction with care, that was derived from two sources [25, 26], coordination with care [26] and level of health service utilisation [27].

The second tool used was the SF-12® survey [28] to measure quality-of-life and functional status. The tool contains 12 items that are used to construct physical component summary (PCS) and the mental component summary (MCS) scores. The SF-12 has demonstrated reliability and validity [29, 30], including in Australia [31, 32]. The instrument has been used previously for investigation of patients with non-cardiac chest pain [33] and for patients managed by the ENP service in Australia [34].

A self-administered questionnaire, using a component of the National Nurse Practitioner Survey [24] was completed by the nurse practitioner in each participating emergency department at the commencement of the study. This tool is an instrument that was used in two previous national nurse practitioner censuses [13, 35]. The tool included items related to barriers and facilitators to practice and the professional (years of experience) and psychosocial (perceived level of competence) characteristics of the nurse practitioner. These data were collected to establish the structural characteristics of the emergency nurse practitioner service.

### Statistical analysis

Sample size calculations were based on the primary outcome for the study and reported in the previously published study protocol [36]. Using the results from our preliminary study [37], we anticipated a six-month period for data collection; however, there were critical issues with participant recruitment necessitating termination of the study prior to achieving the requisite sample. Data analysis was performed according to the pre-published analysis plan [36].

Baseline characteristics were reported separately for each service model. Fisher’s exact test was used to test for significant differences between groups for the dichotomous variables of sex, and regular general practitioner. The independent t-test was used to test for significant differences between groups for the continuous variables of age (mean and standard deviation) and number of emergency department attendance in the previous year (median and IQR). The remaining variables of interest were the categorical variables of Aboriginal or Torres Strait Islander status, employment status, Australasian Triage Score, general practitioner service use, nurse practitioner service use and discharge diagnosis. Chi-square analysis was used to test for significant differences.

Descriptive statistics were used to present data for service indicators, unplanned representation within seven-days and to summarise the adherence to guidelines for patients within the nested cohort. A blinded assessor who has specialist qualifications in emergency medicine performed independent interpretation of electrocardiograms, which was compared to the clinician’s interpretation. Dichotomous data were displayed as a percentage of agreement proportion; comparisons between the service models were examined and tested for significance using Fisher’s exact test. Dichotomous data for unplanned representations were presented as an odds-ratio. Data were compared between service models and tested for significance using the Mann Whitney U test for analysis of service indicators and Fisher’s exact test for analysis of unplanned representations.

We used the Chi-square test and, where appropriate Fisher’s exact test, to compare data for patient satisfaction between service models. In the quality-of-life and functional status assessment, data for the SF-12® summary scores were managed according to the Developer’s guidelines. Data were presented using mean (SD) and were tested for significance using paired t-test. Regression analysis was conducted to evaluate the association between service model and quality-of-life and functional status, adjusted for age and gender. Using the results of this regression, the predicted means for the summary scores at the occasion-of-service and for the follow-up examination for each service model were calculated using the study cohort mean age. These data were compared for each service model and compared for statistical significance using the independent samples t-test.

Data collected to determine the structural characteristics of the nurse practitioner service were presented using descriptive statistics. Due to the nurse practitioner participant small sample size, results were presented as counts in narrative.

All statistical analyses were conducted using de-identified patient data using SPSS (Version 24). The significance level was set at *p* < 0.05.

## Results

**Fig. 1:**
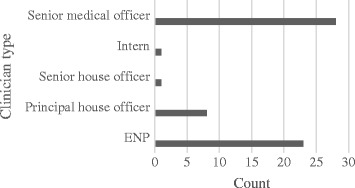
Participant numbers by clinician type

A total of 61 participants were recruited to the study from the three participating sites. Of these 23 (37.7%) participants were managed using the emergency nurse practitioner service model, whilst the remaining 38 (63.3%) were managed using the standard care model (see Fig. 1). Differing levels of experience for the medical officers leading the standard care service were observed, however, many participants (*n* = 28, 73.7%) managed by this service model were reviewed by a senior medical officer (see Fig. 1).

A total of four emergency nurse practitioner participants were recruited to the study from the three participating sites. No nurse practitioner from any site declined to participate in this research.

### Baseline characteristics

Analysis of baseline participant characteristics found no significant difference between the two groups. The age of participants ranged from 20.8 years to 95.7 years. The mean age of participants was 61.0 years (SD 15.5) and 57.4% of the study cohort were female. There were few Aboriginal or Torres Strait Islander participants (*n* = 5, 8.2%). Retired or aged pensioners accounted for more than half (54.1%) of the study cohort. Many of the participants had completed high school or had higher educational qualifications (62.4%). Most participants were allocated either Australasian Triage Score 2 or Australasian Triage Score 3 (92.6%). A clear majority of participants had not previously used a nurse practitioner service (80.3%), reported having a regular general practitioner (91.5%) and attended their general practitioner “every couple of months” (44.3%). The median number of emergency department attendances in the previous year was one (IQR 3).

There were no differences in the baseline characteristics for either service model. Table 1 provides a summary of all patient characteristics.

**Table 1:** Baseline patient characteristics

	Standard care *n* = 38	ENP service *n* = 23	*p* value
Sex			
Male	19	7	
Female	19	16	0.18
Age - years			
Mean (SD)	61.7 (15.4)	59.9 (16.0)	0.66
Aboriginal or Torres Strait Islander status (*n* = 52)			
Not ATSI	25	22	
Aboriginal not TSI	3	1	
TSI not aboriginal	1	0	0.47
Employment status (*n* = 60)			
Employed	11	8	
Pensioner	21	12	
Unemployed	2	0	
Student	1	0	
Home duties	1	2	
Other	1	1	0.67
Highest level of education (*n* = 57)			
Primary school only	16	6	
High school	11	5	
Higher qualifications	9	10	0.21
Australasian Triage Score category (*n* = 54)			
ATS 2	21	5	
ATS 3	13	11	
ATS 4	2	2	0.10
Regular general practitioner (*n* = 59)			
Yes	35	19	
No	2	3	0.35
Emergency department attendances in the past year (*n* = 60)			
Median (IQR)	2 (4)	1 (2)	0.26
General practitioner service use in past year (*n* = 56)			
Not at all	0	1	
Once or twice	13	3	
Every couple of months	14	13	
Once a month	2	2	
More regularly	5	3	0.28
Nurse practitioner service use in past year (*n* = 58)			
Not at all	30	19	
Once or twice	4	3	
Once a month	2	0	0.72

**Fig. 2:**
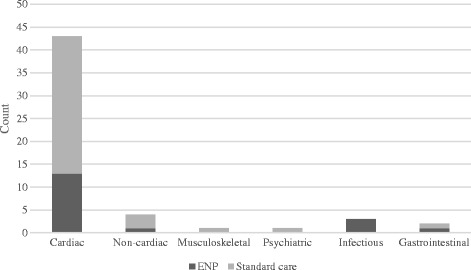
Diagnosis on discharge by condition for each service model

Notably cardiac conditions were implicated in the majority of participants recruited to the study (79.3% of all diagnoses for participants recruited to the study), followed by non-cardiac chest pain (*n* = 4, 7.4%). The remainder of diagnoses included participants with psychiatric, infectious, gastrointestinal and musculoskeletal conditions (see Fig. 2). The single most common discharge diagnosis was “possible cardiac chest pain”, which represented 63.0% (*n* = 34) of all presentations for participants recruited to the study.

Most participants were admitted to the health service at the completion of the emergency department occasion-of-service (77.8%, *n* = 42), nine participants were discharged (16.7%) and the remaining three (5.6%) were transferred to another hospital directly from the emergency. Of the participants admitted to hospital, the majority 63% (*n* = 34) were admitted to the high dependency/short stay unit and the remainder were admitted to the medical unit (14.8%, *n* = 8).

### Adherence to guidelines – Primary outcome

Forty-one participants were identified for inclusion in the nested cohort. The proportion of agreement by service model is presented in Table 2. Although it appeared that the standard care model achieved a higher proportion of adherence to recommendation for timely electrocardiograph review, there was no statistical difference between groups (Fisher’s exact test = 0.11).

**Table 2:** Adherence to guidelines for the nested cohort (suspected or confirmed acute coronary syndrome) by service model - proportion of agreement

	Standard care *n* = 28	ENP service *n* = 13	*p* value
Oxygen, aspirin and pain relief ordered			
*Oxygen administered only to patients with hypoxia (SaO*_*2*_ *< 93%) (n = 40)*			
Compliant	82.1%	100%	
Non-compliant	17.9%	0%	0.20
*Aspirin prescribed in ED (n = 41)*			
Compliant	89.3%	100%	
Non-compliant	10.7%	0%	0.31
12 lead ECG performed and reviewed within 10 min of presentation (*n* = 36)			
Compliant	73.9%	53.8%	
Non-compliant	26.1%	46.2%	0.11
Troponin testing on arrival to ED (*n* = 41)			
Compliant	92.9%	100%	
Non-compliant	7.1%	0%	0.46
Chest x-ray scheduled (*n* = 41)			
Compliant	60.7%	61.5%	
Non-compliant	39.3%	38.5%	0.62
Repeat troponin testing at 6–8 h (*n* = 40)			
Compliant	82.1%	91.7%	
Non-compliant	17.9%	8.3%	0.25
NSTEACS and high-risk patient management			
*Clopidogrel administered in ED (n = 20)*			
Compliant	64.3%	83.3%	
Non-compliant	35.7%	17.7%	0.17
*Enoxaparin administered in ED (n = 19)*			
Compliant	71.4%	80.0%	
Non-compliant	28.6%	20.0%	0.51
STEMI management (*n* = 2)			
*Thrombolysis given if not contraindicated*			
Compliant	100%	100%	
Non-compliant	0%	0%	

### Diagnostic accuracy of electrocardiograph interpretation

**Fig. 3:**
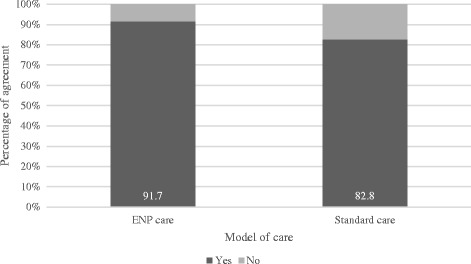
Diagnostic accuracy of ECG interpretation - percentage of agreement by service model

The emergency nurse practitioner model achieved a higher proportion of agreement (91.7%) than the standard care model (82.8%) for diagnostic accuracy of electrocardiograph interpretation (Fisher’s exact test = 0.52) (see Fig. 3).

### Service indicators

For the study cohort, the minimum waiting time was 0 min, whilst the maximum waiting time was 60 min. The mean waiting time was 13.2 min (SD 15.2) and the median waiting time was 8.0 min (IQR 20). Data was not normally distributed (skewness = 1.24, kurtosis = 0.74). The minimum LOS was 23 min, whilst the maximum LOS was 375 min. The mean LOS was 116.0 min (SD 71.6) and the median LOS was 100.0 min (IQR 64). Data were not normally distributed (skewness = 2.12, kurtosis = 5.12). For this study, there was no incidence of participants not waiting to be seen by the care models (Did-not-wait rate = 0%). Table 3 provides a summary of service outcome indicators comparison by service model.

**Table 3:** Service outcome indicators comparison by service model

Outcome	Standard care *Median (IQR)*	ENP model *Median (IQR)*	Difference *Minutes*	*p*-value
Waiting time minutes	7.5 (20)	8 (23)	0.5	0.4
Length of stay minutes	101.5 (54)	97.0 (91)	4.5	0.8

### Diagnostic accuracy as measured by unplanned representation within seven-days

Four participants had an unplanned representation within seven-days. All participants who had an unplanned representation were managed in the standard care model. Although not statistically significant (Fisher’s exact test, *p* = 0.29), participants in this study were 2.4 times more likely to have an unplanned representation if managed by the standard service model.

### Satisfaction with care

At the occasion-of-service all participants were satisfied with the care; 91.5% of participants reported being “highly satisfied” and 8.5% of participants were “satisfied”. There were no differences found between service models.

A high level of rapport between clinicians and participants was demonstrated for both baseline and follow-up measures. All participants reported that they could talk easily and openly and the clinician answered all questions and concerns. There were differences found between service models for the remaining areas of investigation (see Table 4).

**Table 4:** Satisfaction with care - differences between service models

	Standard care model	ENP care model	*p* value
How often did the clinician explain things to you in a way that was easy to understand? (*n* = 60)			
Always	81.1%	78.3%	
Almost always	16.2%	21.7%	
Usually	2.7%		0.65
How often did the clinician listen carefully to you? (*n* = 60)			
Always	86.5%	95.7%	
Almost always	13.5%	4.3%	0.25
Did you feel that the clinician spent enough time with you? (*n* = 58)			
Yes, definitely	97.3%	100%	
Yes, somewhat	2.7%		0.44
Did the clinician tell you in detail about the risks and side effects of the recommended treatment? (*n* = 33)			
Yes, definitely	75.9%	78.6%	
Yes, somewhat	20.7%	14.3%	
No, definitely not	3.4%	7.1%	0.78
Did the clinician give you enough information about treatment choices? (*n* = 24)			
Yes, definitely	90.0%	75.0%	
Yes, somewhat	10.0%	25.0%	0.30
Did the clinician ask which treatment you preferred? (*n* = 21)			
Yes, definitely	78.9%	66.7%	
Yes, somewhat	21.1%	22.2%	
No, definitely not		11.1%	0.33
Did the clinician assist you to make changes in your lifestyle to improve your health or prevent illness? (*n* = 56)			
Yes, definitely	29.3%	13.3%	
Yes, somewhat	12.2%	26.7%	
No, definitely not	2.4%	0%	
No help required	56.1%	60.0%	0.35

At follow-up, there was little change in the levels of participant satisfaction with care, with 93.2% “highly satisfied” and the remaining 6.8% responding that they were “satisfied” with the care they received in the emergency department. There were no significant differences found between service models. All participants reported that they would be “very happy” to reattend the emergency department with chest pain if needed.

### Quality-of-life and functional status

**Fig. 4:**
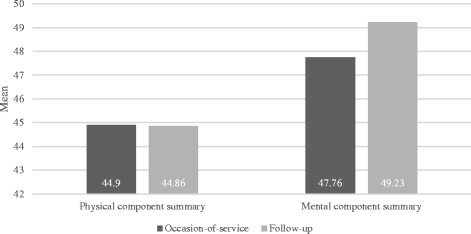
Summary scores for components of SF-12 survey

The mean PCS score for the study cohort did not change significantly (t_(41)_ = 0.51, *p* = 0.96) between the occasion-of-service (44.90, SD 11.6) and follow-up (44.86, SD 11.8). Participants had a change in mean MCS score between the occasion-of-service (47.76, SD 10.7) and follow-up (49.23, SD 10.5), a statistically significant increase of 1.47 (95% CI 0.05 to 3.0), t_(41)_ = 1.96, *p* = 0.05 (see Fig. 4). When adjusted for age and sex, there was no difference between predicted PCS and MCS scores between service models (see Table 5).

**Table 5:** Comparison of predicted means summary scores for SF-12 – adjusted for age and sex

	Standard care Predicted mean	ENP service Predicted mean	*p* value
Physical Component Summary			
Occasion-of-service	44.39	47.49	0.11
Follow-up	44.07	46.98	0.17
Mental Component Summary			
Occasion-of-service	49.58	48.63	0.59
Follow-up	49.16	48.14	0.62

### Structural characteristics of the emergency nurse practitioner service model

- *Professional characteristics (years of experience)*
  The age of participants ranged between 32 and 39 years. The mean age of nurse practitioner participants was 40.5 years (SD 6.95). These nurse practitioners were experienced clinicians, with the mean length of experience as a registered nurse being 15.7 years (SD 4.11). The minimum years of experience was 11 years and the maximum was 21 years. There was considerable variance in the amount of experience as an endorsed nurse practitioner between participants; ranging from 14 to 62 months. The median months of employment as a nurse practitioner was 21 months (IQR 38).
- *Barriers and facilitators for emergency nurse practitioner practice*
  The questionnaire used a series of seven items to assess the barriers and facilitators for nurse practitioner practice. Each item had six possible responses ranging from 1 not at all limiting – 5 extremely limiting. The following items were assessed: (i) lack of Medicare provider number; (ii) support from within the nursing profession; (iii) support from medical colleagues within the health service; (iv) support from colleagues receiving nurse practitioner referral; (v) organisational support; and, (vi) legislative support.
  Data reporting the lack of a Medicare provider number was clearly split between the two extremes for this item; two nurse practitioners reported a lack of Medicare provider number extremely limiting, whilst the other two participants reported this as not at all limiting. Both participants reporting this item as extremely limiting were employed at the same study site.
  In the evaluation of support for the service model there was a range of responses for each item. Most nurse practitioners (*n* = 3) were ambivalent regarding support from within nursing scoring this item a “3” (neither not at all limiting or extremely limiting). The final participant, who had the greatest length of emergency nurse practitioner experience, scored this item a “2”. Similar, for the item “support from medical colleagues” two of the participants scored this item “3”. Interestingly, the final two responses for this item were very dissimilar (a score of “2” and “5”) in the circumstance of both nurse practitioners being employed at the same study site. Two participants reported the service experienced few limitations when referring to colleagues (scoring a “2”), another was ambivalent (“3”) and the remaining nurse practitioner reported extremely limited support. For the two nurse practitioners employed at the same study site, organisational support was not limiting or facilitating (scoring a “3”) to the service model. The remaining two participants reported extremely limited organisation support (scoring a “5”) for the service model. Lastly, notwithstanding all nurse practitioners being employed under the same legislative conditions, there was limited agreement between the participants in the evaluation of legislative support for the model. There was also no significant evidence toward either the existence or lack of legislative support; two scores of “3”, one of each “2” and “4” were provided.
- *Psychosocial characteristics (perceived level of competence)*
  The highest score for this item was a “2” (indicating a high level of perceived competence) was reported by a single participant who also possessed the greatest level of experience as a nurse practitioner. The remaining participants (*n* = 3) were ambivalent about their self-reported competence.

## Discussion

This study lends support to the evidence of effectiveness of the emergency nurse practitioner service model when compared to the standard care model in the provision of care to rural patients with complex health care needs. Although problems with patient recruitment ultimately led to an underpowered study, due to the paucity of research in this field it is important that these study results are available for potential subsequent meta-analysis in reviews or studies evaluating the effectiveness of the emergency nurse practitioner service model.

### Adherence to guidelines

Clinical guidelines have the fundamental goal of improving the quality and safety of health care by ensuring evidence based practice and reducing variations in health care [38]. Despite this, there are often gaps between the guideline recommendations and clinical practice, that may result in patients not receiving appropriate care [39, 40]. Few studies have examined the extent of adherence to clinical guidelines in the emergency environment [41]. Consequently, little is known about guideline use in the Australian context and in the rural setting. Previous evaluation has found 38% adherence to guidelines for the management of adult patients presenting to an Australian metropolitan emergency department with asthma [42]. The National Heart Foundation and Cardiac Society of Australia and New Zealand guidelines [43, 44] provide recommendations on the risk stratification and management of patients presenting to emergency departments with suspected or confirmed acute coronary syndrome. This is the first study to examine the extent to which these guidelines are followed for the cohort of patients presenting to rural emergency departments with chest pain. Overall, adherence to the guidelines by clinicians in this study was good with clinicians achieving a minimum of 64% compliance with acute coronary syndrome guidelines.

Oxygen appeared to be overused by the standard care model. The guidelines recommend oxygen be administered to those with hypoxia (SpO_2_ less than 94%) or signs of shock. For this study, non-compliance occurred when oxygen was administered when not clinically indicated. A recent systematic review [45] found no conclusive evidence to support the routine use of supplemental oxygen and suggested that there may be an increased risk of death to patients with acute coronary syndrome who received oxygen.

A high proportion of participants (greater than 82%) were administered aspirin in our study. This compares well with a 2007 cross-sectional study of 544 emergency departments in the USA that aimed to evaluate the proportion of patients receiving guideline recommended care in acute coronary syndrome and found that aspirin was administered to only 40% of patients in the study [46].

In the same way, a high proportion of participants in our study (greater than 82%) had cardiac biomarker testing performed on arrival to emergency department and repeated at the guideline recommended interval. On the other hand, there was suboptimal adherence to 12-lead electrocardiograph review within 10 min of presentation (53%) and chest x-ray scheduling (60.7%). Although two-thirds of electrocardiographs performed were in accordance with guidelines, there was an inappropriately high proportion of patients that did not have timely review of their electrocardiograph. It is possible that non-adherence to guideline regarding chest x-ray scheduling may have occurred because of barriers specific to the rural environment. For the participating sites in this study radiology services are on-call after hours, which may have led to clinicians appropriately rationing resources and not performing routine x-ray investigations on clinically well patients.

Our results show that there was a higher proportion of guideline adherence for high-risk patients and those with diagnosed acute coronary syndrome by the emergency nurse practitioner service model. The guideline adherence rate was greater than 80% for administration of both clopidogrel and enoxaparin, whilst the standard care model achieved 64.3% and 71.4% respectively. Furthermore, although the standard care model achieved a good level of adherence to the guidelines, the proportion of patients receiving guideline-recommended care was lower than that achieved by the emergency nurse practitioner service model. The reason for this may be that the medical officers studied may have preferred to exercise professional autonomy in making clinical judgements based on personal experience, which has been found to influence adherence to emergency department guidelines [47].

### Diagnostic accuracy of electrocardiograph interpretation

Whilst research has found that emergency nurse practitioner service achieves high diagnostic accuracy, previous investigations have been primarily conducted in the area of minor injury and illness with studies reporting on missed injury and fracture rates [48–50]. This study is the first to evaluate the diagnostic accuracy of emergency nurse practitioner service in complex investigation. This care model achieved a higher level of diagnostic accuracy of electrocardiograph interpretation (91.7%) than the standard care model (82.8%). Whilst there is no single or combination of clinical features that can be used to exclude acute coronary syndrome, the initial evaluation and management of a patient with undifferentiated chest pain requires a meticulous clinical assessment with interpretation of electrocardiograph being the cornerstone of the assessment [22]. A missed diagnosis of acute coronary syndrome may result in a delay in initiating the appropriate treatment and increasing the mortality rate [51, 52]. In the context of the findings of this study, there was the potential for nearly one-fifth of all patients presenting with undifferentiated chest pain to be exposed to significant risk from either a missed opportunity for intervention or from further unnecessary testing and intervention.

### Service indicators

This study found no significant difference between the two clinician groups with regard to waiting times, length-of-stay and did-not-wait times. This finding is consistent with the most recent research that has evaluated emergency nurse practitioner service on these indicators in Australian emergency departments [53]. Although other studies have demonstrated a reduction in the length-of-stay for patients managed by an emergency nurse practitioner service [54, 55], these findings are limited because there was no standardised definition for the clinician groups studied. In these studies [54, 55], doctors with lower levels of experience required “sign-off” by a senior colleague and there were marked differences in the responsibilities of these clinicians whilst the emergency nurse practitioner comparator had lower levels of interruption with a clear focus on the management of minor injury and illness. Furthermore, many of these studies were based on retrospective audit data rather than prospective studies [56–58]. Our prospective cohort study avoided these limitations and the majority of patients that were managed in the standard care model had a senior medical officer as the lead clinician.

### Diagnostic accuracy as measured by unplanned representation within seven-days

The overall unplanned representation rate for patients presenting to rural hospitals with undifferentiated chest pain was 6.6%, higher than the 0.6% rate previously reported [37].In our study patients were more than twice as likely to have an unplanned representation within seven-days if they were managed in the standard care model. Of these half represented with chest pain Whilst studies have demonstrated no difference between clinician groups [34, 54] in unplanned representation rates other studies support our findings [59, 60] As previously indicated, the majority of patients who present to emergency department with undifferentiated chest pain will have no cardiac cause and will be ultimately discharged with a diagnosis of non-cardiac chest pain. This patient cohort has been found to have increased anxiety, reduced quality-of-life, further chest pain and an increased demand for health care services [61–63].

### Satisfaction with care

The acceptability of the emergency nurse practitioner service has been clearly established with consistently high levels of patient satisfaction reported in the literature [11, 64–70]. Our study supports this evidence in finding the majority (88.5%) of participants were highly satisfied with the overall quality of care, which was sustained over time. At the follow-up evaluation, 93.2% of participants reported that they were highly satisfied with the overall quality of care. Whilst previous studies [34, 64, 66, 67] have found higher levels of patient satisfaction with emergency nurse practitioner service when compared to the standard care model, our study did not demonstrate any significant difference between models.

The entirety of participants from both groups reported that the clinician seemed informed and up-to-date and the majority reported that the clinician assisted them to make lifestyle changes to improve their health. Pursuing this further, participants in our study had a high level of rapport with clinicians. The majority reported that they could discuss their concerns and be listened to carefully, explanations by the clinician were easily understood and participants felt involved in their health care decision making. In the same way Jeanmonod, et al. (2013) also found that the majority of patients of an emergency nurse practitioner service felt cared about, were kept aware of tests and had their problems and follow-up explained. In addition, our study found that high levels of satisfaction with care were maintained at follow-up evaluation.

### Quality-of-life and functional status

Participants in our study were found to have change in the MCS summary score at follow-up measurement (+1.47). Whilst statistically significant (*p* = 0.05), this is not clinically relevant and unlikely to represent an improvement as a result of service intervention. There were no differences between participants from either service model.

The mean PCS and MCS score when adjusted for age and sex for Australian adults with heart disease had previously been recorded as 44.4 and 50.2 respectively [71]. Similarly, a mean PCS of 40.90 (SD 11.7) and MCS of 49.14 (SD 10.9) has been reported for patients with ACS [72]. The mean summary scores for the SF-12 for our study cohort were comparable to these findings.

### Structural characteristics of the emergency nurse practitioner service model

The nurse practitioners in our cohort are considerably younger and less experienced as registered nurses than those studied in the last national census of Australian nurse practitioners [13]. The mean age of nurse practitioners in our study was 40.5 years and the length of time employed as a registered nurse was 15.7 years. The rural context itself most likely provides explanation for this finding, by providing opportunity for early career development for its nurses. Faced with crucial medical workforce issues [73] and rural hospitals struggling to maintain health services [74], nurses have played a vital role in the delivery of services. The existing rural health service culture has a reliance on rural nurses to be multi-skilled generalists with a wide range of advanced skills [75], often making clinical decisions in the absence of other health professionals [76]. In Australia, registered nurses who seek endorsement as nurse practitioners are required to complete a Master’s degree. Students applying to these courses must demonstrate a minimum of three years of experience working at an advanced practice level. The development and utilisation of emergency nurse practitioner models of care in rural health service represents a natural progression for these career rural nurses.

No specific rural health service structural characteristics were reported the nurse practitioner cohort as being barriers or facilitators to the service model. Results from the two national nurse practitioner censuses [13, 35] concluded that the majority of Australian nurse practitioners reported significant barriers to practice, with concerns about the capacity to care for patients to the full extent of the role noted [35]. Other research, more specific to the rural context of emergency nurse practitioner practice, demonstrated that a lack of support from the organisation and colleagues was a barrier to senior nurses considering endorsement [77]. These concerns were not supported by the results of our study. Not-with-standing the challenges, the rural environment presents many opportunities for innovation, including the use of emergency nurse practitioner service. The nurse practitioners surveyed did not express concerns regarding a lack of support from nursing or medical colleagues, legislation or organisational support.

Evaluating self-perceived competence provides an indication of the individual’s motivation in maintaining and improving skills [78] and is a component of self-efficacy, one’s belief in their ability to succeed in specific situations or accomplish a task. Despite the rural setting providing preparation for extensions to nursing practice, the participants in our study do not perceive themselves as either being limited or not limited by their self-perceived role competence. This finding was concerning; previous knowledge has suggested that a professionals’ self-efficacy plays an important role in overall job performance [79] and further, in the case of low self-efficacy, practice could fall below evidence based recommendations [80]. Unlike their metropolitan counterparts, in rural emergency departments nurse practitioners must work to the full scope of their expanded role across all patient acuities [81] including those presenting with complex conditions including chest pain. This “generalist” practice may provide explanation as to our nurse practitioners ambivalence regarding self-perceived competence. In metropolitan hospitals, health services are generally provided by dedicated specialist staff, with the emergency nurse practitioner service model centred on the delivery of care to patients with minor injury or illness. Dissimilarly, the rural service is required to provide care to patients that encompasses a wide variety of diagnoses. The rural emergency nurse practitioner service is required to have wide knowledge and skills to deliver safe and effective care that may preclude a mastery in any area and thus, impact on reported self-perceived competence.

### Strengths and limitations of the study

This was an observational study. Although a randomised controlled trial would have met the “gold standard” for research, in this case it was not feasible to implement emergency nurse practitioner clinicians as a service intervention. The service was already established and hence an observational study was conducted consistent with established practice to evaluate the quality of patient and service outcomes.

The major limitation of our study is the small sample size that has led to an inability to compare the safety and quality of care for the service models with statistical significance. Whilst initial estimates of presentations in the participating hospital indicated sufficient numbers for our sample size estimates, requisite patient recruitment did not proceed as anticipated. Although this study was underpowered to confirm the study hypotheses, the results were significant. Data from underpowered studies that use methodological rigor to eliminate bias and are accurately reported should be made routinely available [82–84]. Furthermore, in this case these results play an important role as foundation evidence for the evaluation of the emergency nurse practitioner service in a previously unknown context, with an unbiased study with imprecise results far better than no results at all [84]. The issues with patient recruitment may have also introduced selection bias; despite a standardised protocol patient recruitment varied across sites.

Our study benefits from rigorous research methods and the use of an appropriate study design. We used a suite of validated and well tested tools to evaluate the multiple dimensions of the emergency nurse practitioner role, including the substance of nursing care and its influence on patient outcomes. In assessing the diagnostic accuracy of electrocardiograph interpretation, we used an emergency consultant (the “gold standard”) for blinded assessment of electrocardiographs. The study design avoided the limitations of previous emergency nurse practitioner research by using qualified emergency nurse practitioners working to the full scope of their role and a standardised comparator.

## Conclusion

The MaP-RED study is the first reported study that has examined the effectiveness of emergency nurse practitioner service in the management of patients presenting to rural EDs with chest pain. Our study found a high level of adherence to clinical guidelines for the emergency nurse practitioner service model and a concomitant high level of diagnostic accuracy. In evaluation of the service indicators of waiting time and length-of-stay the emergency nurse practitioner service demonstrated comparable effectiveness to that of the standard care model. In addition, excellent patient reported outcomes for the emergency nurse practitioner service model were demonstrated.

These findings provide a foundation for the beginning evaluation of rural emergency nurse practitioner service in the delivery of safe and effective care beyond the minor illness and injury cohort.

## Additional files

Additional file 1: Patient participant questionnaire – Baseline. (PDF 163 kb)

Additional file 2: Patient participant questionnaire – 30-day follow-up. (PDF 137 kb)

## Abbreviations

ACS: Acute coronary syndrome
ED: Emergency department
ENP: Emergency nurse practitioner
NP: Nurse practitioner
