# Supplementary material for: The effectiveness of emergency nurse practitioner service in the management of patients presenting to rural hospitals with chest pain: a multisite prospective longitudinal nested cohort study
Source: BMC Health Serv Res. 2017 Jun 27;17:445. doi: 10.1186/s12913-017-2395-9 (PMC5488347; doi:10.1186/s12913-017-2395-9)
Supplement: Supplementary file 1 — Patient participant questionnaire – Baseline. (PDF 163 kb) [file 12913_2017_2395_MOESM1_ESM.pdf]

Queensland University of Technology
Brisbane Australia

Managing chest pain in rural emergency departments

Participant Questionnaire

DEMOGRAPHICS

The first questions are general questions about you.

1. What is your sex?

Male ☐1 Female ☐2

2. What is your date of birth?

D D M M Y Y Y Y
☐☐☐☐☐☐☐☐

3. Do you identify as Aboriginal or Torres Strait Islander?

Neither Aboriginal nor Torres Strait Islander………….. ☐1
Aboriginal not Torres Strait Islander………………….... ☐2
Torres Strait Islander not Aboriginal ………………...... ☐3
Aboriginal and Torres Strait Islander …...…………….. ☐4

4. What is your employment status?

Employed full-time …………………................. ☐1
Employed part-time ……………….................... ☐2
Unemployed ………………...………………… ☐3
Invalid pensioner...…………………………….... ☐4
Aged pensioner / retired……………………….. ☐5
Student ………………………………………… ☐6
Home Duties………………………………….... ☐7
Other …………………………………………... ☐8

5. What is the highest level of education you have achieved?

Did not complete primary school …...................☐1
Primary school only........………………………☐2
No intermediate or school certificate…………..☐3
Leaving or higher school certificate…...……… ☐4
TAFE ……………………………………….....☐5
College...………………………………………..☐6
University...……………………………………...☐7

6. Which of the following health services have you used in the last year?

	GP	Community Health	Specialist medical officer	Allied Health	Nurse practitioner
Not at all					
Once or twice					
Every couple of months					
Once a month					
More regularly					

7. Do you have a regular General Practitioner?

Yes……………... ☐1
No ………………☐2

8. In the past year, how many times did you go to an Emergency Department?

……………………………………………………......……………………………………………

9. In the past year, how many times did you stay overnight or longer in a hospital?

………………………………………………………………....…………………………………

CHEST PAIN MANAGEMENT

The next questions are about the chest pain that you experienced in the emergency department and how you feel the doctor or nurse practitioner managed the pain.

10. Did you experience any chest pain while you were being treated in the emergency department?

Yes …………….. ☐1 go to Q 11
No ……………… ☐2 **go to Q 16**

11. If yes, could you please indicate the severity of this pain?

Mild	Moderate	Severe
☐1	☐2	☐3

12. How long was the pain present for?

All of the time	Most of the time	Some of the time	Only occasionally
☐1	☐2	☐3	☐4

13. Was the doctor/nurse practitioner aware of this pain?

Yes …………….. ☐1
No ……………... ☐2

14. Were you offered treatment for this pain?

Yes…………….. ☐1 go to Q 15
No……………..... ☐2 **go to Q 16**

15.If yes, how satisfied were you with this treatment?

Highly satisfied	Satisfied	Neither satisfied nor dissatisfied	Dissatisfied	Highly unsatisfied
☐1	☐2	☐3	☐4	☐5

SATISFACTION WITH EMERGENCY CARE

The next questions are about how you feel about the care that you received for your chest pain. Please read the questions carefully, keeping in mind the care that you have just received in the emergency department. We are interested in your experiences, good and bad, about the care that you have received.

16.Did the doctor/nurse practitioner seem informed and up-to-date about the care you were provided?

Yes, always	Yes, sometimes	No, never
☐1	☐2	☐3

17.When the doctor/nurse practitioner performed a blood test, x-ray or other test, did they follow-up to give you the test results?

Yes, always	Yes, sometimes	No, never	I did not have any tests
☐1	☐2	☐3	☐4

18.Have your reasons for presenting to the emergency department been sorted out/resolved?

In full	In part	Not at all
☐1	☐2	☐3

If only in part or not at all, please could you tell me about this?

……………………………………………………………………………………………

……………………………………………………………………………………………

……………………………………………………………………………………………

……………………………………………………………………………………………

19. Were you satisfied with the doctor/nurse practitioner's treatment of your chest pain?

Highly satisfied	Satisfied	Neither satisfied nor dissatisfied	Dissatisfied	Highly unsatisfied
☐1	☐2	☐3	☐4	☐5

20. How often did the doctor/nurse practitioner <u>explain things</u> in a way that was easy to understand?

Never	Almost never	Sometimes	Usually	Almost always	Always
☐1	☐2	☐3	☐4	☐5	☐6

21. How often did the doctor/nurse practitioner <u>listen carefully</u> to you?

Never	Almost never	Sometimes	Usually	Almost always	Always
☐1	☐2	☐3	☐4	☐5	☐6

22. Did you feel that you could talk to the doctor/nurse practitioner easily and openly?

Yes, definitely	Yes, somewhat	No, definitely not
☐1	☐2	☐3

23. Did you feel that the doctor/nurse practitioner spent enough time with you?

Yes, definitely	Yes, somewhat	No, definitely not
☐1	☐2	☐3

24. Did you feel that the doctor/nurse practitioner answered all your questions and concerns?

Yes, definitely	Yes, somewhat	No, definitely not
☐1	☐2	☐3

25. Did the doctor/nurse practitioner recommend a treatment for the chest pain?

Yes…………….. ☐1 go to Q 26
No ………………☐2 **go to Q 31**

26. Did the doctor/nurse practitioner tell you in detail about the risks and side effects of the recommended treatment?

Yes, definitely	Yes, somewhat	No, definitely not
☐1	☐2	☐3

27. Did the doctor/nurse practitioner say that there was <u>more than one treatment option</u> to consider for your care?

Yes……………..… ☐1 go to Q 28
No………………☐2 **go to Q 31**

28. When there was more than one treatment option to consider, did the doctor/nurse practitioner give you <u>enough information</u> about each option?

Yes, definitely	Yes, somewhat	No, definitely not
☐1	☐2	☐3

Queensland University of Technology
Brisbane Australia

29. When there was more than one treatment option to consider, did the doctor/nurse practitioner ask which treatment you preferred?

Yes, definitely	Yes, somewhat	No, definitely not
☐1	☐2	☐3

30. Did the doctor/nurse practitioner give you the help you need to make changes in your habits or lifestyle that would improve your health or prevent illness?

Yes, definitely	Yes, somewhat	No, definitely not	I do not need help with this
☐1	☐2	☐3	☐4

31. Overall, how would you rate the quality of care provided by the doctor/nurse practitioner?

Highly satisfied	Satisfied	Neither satisfied nor dissatisfied	Dissatisfied	Highly unsatisfied
☐1	☐2	☐3	☐4	☐5

SF-12®

This questionnaire asks for your views about your health. This information will help keep track of how you feel and how well you are able to do your usual activities.

For each of the following questions, please mark an ☒ in the one box that best describes your answer.

32. In general, would you say your health is:

Excellent	Very good	Good	Fair	Poor
▼	▼	▼	▼	▼
☐ 1	☐ 2	☐ 3	☐ 4	☐ 5

33. The following questions are about activities you might do during a typical day. Does <u>your health now limit you</u> in these activities? If so, how much?

	Yes, limited a lot	Yes, limited a little	No, not limited at all
a <u>Moderate activities</u>, such as moving a table, pushing a vacuum cleaner, bowling, or playing golf	☐ 1	☐ 2	☐ 3
b Climbing <u>several</u> flights of stairs	☐ 1	☐ 2	☐ 3

34. During the <u>past 4 weeks</u>, how much of the time have you had any of the following problems with your work or other regular daily activities <u>as a result of your physical health</u>?

	All of the time	Most of the time	Some of the time	A little of the time	None of the time
a <u>Accomplished less</u> than you would like	☐ 1	☐ 2	☐ 3	☐ 4	☐ 5
b Were limited in the <u>kind</u> of work or other activities	☐ 1	☐ 2	☐ 3	☐ 4	☐ 5

35. During the <u>past 4 weeks</u>, how much of the time have you had any of the following problems with your work or other regular daily activities <u>as a result of any emotional problems</u> (such as feeling depressed or anxious)?

	All of the time	Most of the time	Some of the time	A little of the time	None of the time
a <u>Accomplished less</u> than you would like	☐ 1	☐ 2	☐ 3	☐ 4	☐ 5
b Did work or other activities <u>less carefully than usual</u>	☐ 1	☐ 2	☐ 3	☐ 4	☐ 5

36. During the <u>past 4 weeks</u>, how much did <u>pain</u> interfere with your normal work (including both work outside the home and housework)?

Not at all	A little bit	Moderately	Quite a bit	Extremely
☐ 1	☐ 2	☐ 3	☐ 4	☐ 5

37. These questions are about how you feel and how things have been with you <u>during the past 4 weeks</u>. For each question, please give the one answer that comes closest to the way you have been feeling. How much of the time during the <u>past 4 weeks</u>…

	All of the time	Most of the time	Some of the time	A little of the time	None of the time
a Have you felt calm and peaceful?	☐ 1	☐ 2	☐ 3	☐ 4	☐ 5
b Did you have a lot of energy?	☐ 1	☐ 2	☐ 3	☐ 4	☐ 5
c Have you felt downhearted and depressed?	☐ 1	☐ 2	☐ 3	☐ 4	☐ 5

38. During the <u>past 4 weeks</u>, how much of the time has your <u>physical health or emotional problems</u> interfered with your social activities (like visiting with friends, relatives, etc.)?

All of the time	Most of the time	Some of the time	A little of the time	None of the time
☐ 1	☐ 2	☐ 3	☐ 4	☐ 5

YOU HAVE FINISHED. THANK YOU FOR YOUR TIME.

Please place the questionnaire in the **supplied envelope** and return the Research Assistant who provided this questionnaire to you.
