# Supplementary material for: The effectiveness of emergency nurse practitioner service in the management of patients presenting to rural hospitals with chest pain: a multisite prospective longitudinal nested cohort study
Source: BMC Health Serv Res. 2017 Jun 27;17:445. doi: 10.1186/s12913-017-2395-9 (PMC5488347; doi:10.1186/s12913-017-2395-9)
Supplement: Supplementary file 2 — Patient participant questionnaire – 30-day follow-up. (PDF 137 kb) [file 12913_2017_2395_MOESM2_ESM.pdf]

Queensland University of Technology
Brisbane Australia

# Managing chest pain in rural emergency departments

## Follow-up Participant Questionnaire

**EMERGENCY DEPARTMENT PRESENTATION**

The following questions relate to your visit to the emergency department because of the chest pain that you experienced last month. Please read the questions carefully, keeping in mind the care that you received in the emergency department on that day. We are interested in your experiences, good and bad, of the care that you received.

1. **Were you satisfied with the doctor/nurse practitioner's treatment of your chest pain?** (Please tick the box that applies)

| Highly satisfied | Satisfied | Neither satisfied nor dissatisfied | Dissatisfied | Highly unsatisfied |
|---|---|---|---|---|
| ☐1 | ☐2 | ☐3 | ☐4 | ☐5 |

2. **Did you feel that the doctor/nurse practitioner spent enough time with you?**

| Yes, definitely | Yes, somewhat | No, definitely not |
|---|---|---|
| ☐1 | ☐2 | ☐3 |

3. **Did you feel that the doctor/nurse practitioner answered all your questions and concerns?**

| Yes, definitely | Yes, somewhat | No, definitely not |
|---|---|---|
| ☐1 | ☐2 | ☐3 |

4. **Overall, how would you rate the quality of care provided by the doctor/nurse practitioner?**

| Highly satisfied | Satisfied | Neither satisfied nor dissatisfied | Dissatisfied | Highly unsatisfied |
|---|---|---|---|---|
| ☐1 | ☐2 | ☐3 | ☐4 | ☐5 |

**5. Would you be happy to reattend the emergency department with chest pain should the need arise?**

| Very happy | Fairly happy | Happy | Fairly unhappy | Unhappy |
|---|---|---|---|---|
| ☐1 | ☐2 | ☐3 | ☐4 | ☐5 |

The following questions are to determine if your chest pain has resolved or not since you were treated in the emergency department.

**6. Have you experienced any further chest pain since the doctor/nurse practitioner treated you in the emergency department last month?**

Yes.…………….. ☐1 **go to Q 7**

No ……………… ☐2 **go to Q 10**

**7. If yes, could you please indicate the severity of this pain?**

| Mild | Moderate | Severe |
|---|---|---|
| ☐1 | ☐2 | ☐3 |

**8. In the last 30-days, how many times did you go to an Emergency Department because of chest pain?**

…………………………...……………………………………………………………………….

**9. In the last 30-days, how many times did you stay overnight or longer in a hospital because of chest pain?**

…………………………...……………………………………………………………………….

**10. Have you used any of the following services because of your chest pain since you were treated last month?**

| | Emergency department | General practitioner | Specialist medical officer | Community Health/Allied Health | Nurse practitioner |
|---|---|---|---|---|---|
| Not at all | | | | | |
| Once or twice | | | | | |
| More than twice | | | | | |

**SF-12®**

This questionnaire asks for your views about your health. This information will help keep track of how you feel and how well you are able to do your usual activities.

For each of the following questions, please mark an ☒ in the one box that best describes your answer.

**11. In general, would you say your health is:**

| Excellent | Very good | Good | Fair | Poor |
|---|---|---|---|---|
| ☐ 1 | ☐ 2 | ☐ 3 | ☐ 4 | ☐ 5 |

**12. The following questions are about activities you might do during a typical day. Does <u>your health now limit you</u> in these activities? If so, how much?**

| | | Yes, limited a lot | Yes, limited a little | No, not limited at all |
|---|---|---|---|---|
| a | <u>Moderate activities</u>, such as moving a table, pushing a vacuum cleaner, bowling, or playing golf | ☐ 1 | ☐ 2 | ☐ 3 |
| b | Climbing <u>several</u> flights of stairs | ☐ 1 | ☐ 2 | ☐ 3 |

**13.During the past 4 weeks, how much of the time have you had any of the following problems with your work or other regular daily activities as a result of your physical health?**

| | All of the time | Most of the time | Some of the time | A little of the time | None of the time |
|---|---|---|---|---|---|
| a Accomplished less than you would like | ☐ 1 | ☐ 2 | ☐ 3 | ☐ 4 | ☐ 5 |
| b Were limited in the kind of work or other activities | ☐ 1 | ☐ 2 | ☐ 3 | ☐ 4 | ☐ 5 |

**14.During the past 4 weeks, how much of the time have you had any of the following problems with your work or other regular daily activities as a result of any emotional problems (such as feeling depressed or anxious)?**

| | All of the time | Most of the time | Some of the time | A little of the time | None of the time |
|---|---|---|---|---|---|
| a Accomplished less than you would like | ☐ 1 | ☐ 2 | ☐ 3 | ☐ 4 | ☐ 5 |
| b Did work or other activities less carefully than usual | ☐ 1 | ☐ 2 | ☐ 3 | ☐ 4 | ☐ 5 |

**15. During the past 4 weeks, how much did pain interfere with your normal work (including both work outside the home and housework)?**

| Not at all | A little bit | Moderately | Quite a bit | Extremely |
|---|---|---|---|---|
| ☐ 1 | ☐ 2 | ☐ 3 | ☐ 4 | ☐ 5 |

Queensland University of Technology
Brisbane Australia

**16. These questions are about how you feel and how things have been with you <u>during the past 4 weeks</u>. For each question, please give the one answer that comes closest to the way you have been feeling. How much of the time during the <u>past 4 weeks</u>…**

| | All of the time | Most of the time | Some of the time | A little of the time | None of the time |
|---|---|---|---|---|---|
| a Have you felt calm and peaceful? | ☐ 1 | ☐ 2 | ☐ 3 | ☐ 4 | ☐ 5 |
| b Did you have a lot of energy? | ☐ 1 | ☐ 2 | ☐ 3 | ☐ 4 | ☐ 5 |
| c Have you felt downhearted and depressed? | ☐ 1 | ☐ 2 | ☐ 3 | ☐ 4 | ☐ 5 |

**17. During the <u>past 4 weeks</u>, how much of the time has your <u>physical health or emotional problems</u> interfered with your social activities (like visiting with friends, relatives, etc.)?**

| All of the time | Most of the time | Some of the time | A little of the time | None of the time |
|---|---|---|---|---|
| ☐ 1 | ☐ 2 | ☐ 3 | ☐ 4 | ☐ 5 |

**YOU HAVE FINISHED. THANK YOU FOR YOUR TIME.**

Please return the questionnaire in the **reply paid envelope supplied** to:

Tina Roche
Emergency Department
Stanthorpe Health Services
PO Box 273
STANTHORPE QLD 4380
